# Is pancreatitis associated with meglumine antimoniate treatment for canine leishmaniosis? A multicentric prospective study

**DOI:** 10.1186/s13071-024-06617-7

**Published:** 2024-12-23

**Authors:** Clàudia Viñeta, Jorge Castro, María Cristina López, Maria Frau, Antón Costas, Carolina Arenas, Xavier Roura

**Affiliations:** 1 Hospital Clínic Veterinari, Universitat Autònoma de Barcelona, Bellaterra, Spain; 2 AniCura Valencia Sur Hospital Veterinario, Valencia, Spain

**Keywords:** Pancreas, Leishmania, Dog, Antimonials

## Abstract

**Background:**

Meglumine antimoniate is used to treat canine leishmaniosis. In humans, it has been associated with pancreatitis. Although a few case reports have described acute pancreatitis secondary to antimonial treatment in dogs, some studies have concluded that pancreatitis is not an adverse effect of this medication. The objective was to evaluate whether treatment with meglumine antimoniate could induce pancreatitis in dogs with leishmaniosis, on the basis of clinical signs, canine serum specific quantitative pancreatic lipase immunoreactivity (cPLI) concentration, and ultrasonographic abnormalities.

**Methods:**

A prospective, observational, longitudinal, and multicentric study was conducted from April 2021 through February 2023.

**Results:**

A total of 33 dogs with leishmaniosis were included and classified into LeishVet clinical stages; 13 (39.4%) were included in stage II, 11 (33.3%) in stage III, and 9 in stage IV (27.3%). and 14 (42.4%) developed pancreatitis, 10 during treatment with meglumine antimoniate, and 4 at the end of the treatment. Advanced LeishVet clinical stage was statistically associated with development of pancreatitis. In addition, nine dogs received prednisone at the beginning of treatment, but it was not statistically associated with the prevention of pancreatitis.

**Conclusions:**

Meglumine antimoniate remains the first line leishmanicidal treatment option for canine leishmaniosis, but it appears to induce pancreatitis in a significant percentage of dogs. Monitoring serum cPLI levels and performing an abdominal ultrasound should be considered when pancreatitis-associated clinical signs are observed, or when there is a high suspicion of circulating immune complexes in dogs with advanced LeishVet clinical stage.

**Graphical Abstract:**

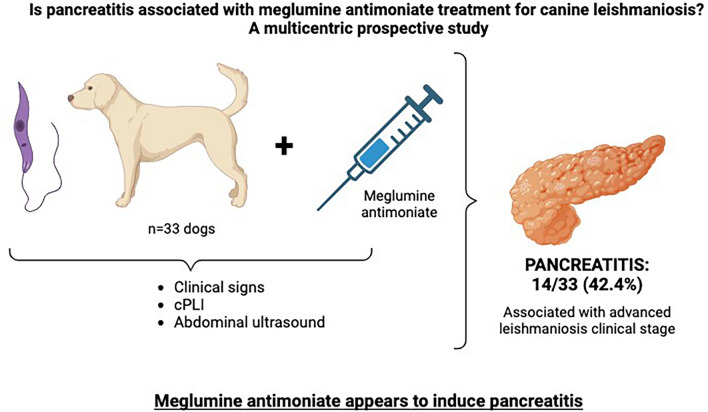

## Background

Canine leishmaniosis is caused by *Leishmania infantum*, a parasitic protozoan transmitted by the bite of phlebotomine sandflies [1]. The combination of *N*-methyl-glucamine (meglumine antimoniate) and allopurinol is considered the first-line and most efficacious treatment for canine leishmaniosis [2, 3].

The most used pentavalent antimonial compound for treating leishmaniosis in dogs and humans is meglumine antimoniate [3]. Pain and swelling at the injection site are the most frequent adverse effects described in dogs, while fever, diarrhea, loss of appetite, and kidney disease have also been reported [3–7]. However, kidney disease is now thought to be a collateral consequence rather than a direct adverse effect, due to the formation and deposition of immune complexes in the kidney [8]. Allopurinol has also been associated with several adverse effects, mainly urinary, including xanthinuria, kidney mineralization, and urolithiasis [9, 10]. Dermatological adverse effects are less commonly reported [11, 12], but there are no reports on pancreatitis associated with allopurinol treatment in either human or veterinary medicine.

In human medicine, pentavalent antimonials, such as meglumine and stibogluconate, have been linked to causing pancreatitis when used to treat visceral leishmaniosis [13–15]. These drugs rank as the fourth most common cause of drug-induced pancreatitis in medical literature [16, 17]. Elevated serum levels of pancreatic enzymes were observed in nearly all human patients treated with pentavalent antimonials, and overt pancreatitis developed in up to 70% of these patients [13].

Pancreatitis caused by meglumine antimoniate treatment has been reported in dogs [7, 18–22]. However, a study found no evidence of a link between meglumine antimoniate use and pancreatitis in dogs with leishmaniosis [23].

Canine pancreatitis is an idiopathic condition with several potential risk factors such as breed, genetic and sex associations, hypertriglyceridemia, obesity, diet, infections, intoxications, and endocrine disorders [24, 25]. Clinical signs may vary between patients and are often nonspecific including apathy, anorexia, weakness, vomiting, diarrhea, melena, abdominal pain, weight loss, hematemesis, hematochezia or polyuria, and polydipsia [26, 27]. On top of that, the diagnosis of canine pancreatitis is challenging for veterinary practitioners because the gold standard for diagnosis is the identification of consistent inflammatory patterns on pancreatic biopsy [28]. However, due to its invasive nature and limitations, including the potential to miss localized lesions, pancreatic biopsy is rarely performed [26, 29, 30]. Therefore, a diagnosis of clinical pancreatitis is often made using a combination of appropriate clinical signs, positive pancreatitis-specific laboratory tests, and abdominal ultrasound findings, as well as excluding other potential diseases with similar clinical presentation [26].

The objective of this study was to evaluate whether treatment with meglumine antimoniate could induce pancreatitis in dogs with leishmaniosis, on the basis of clinical signs, canine-specific quantitative pancreatic lipase immunoreactivity (cPLI) serum concentration, and ultrasonographic abnormalities.

The main hypothesis was that (i) meglumine antimoniate could induce pancreatitis in *Leishmania infantum*-sick dogs. Secondary hypotheses were that (ii) advanced LeishVet clinical stages could increase the risk of developing pancreatitis and (iii) the use of prednisone may have a protective effect reducing the risk of developing pancreatitis.

## Methods

### Animals, inclusion, and exclusion criteria

A prospective, observational, longitudinal, and multicentric study was conducted from April 2021 through February 2023 at different hospitals in Spain. A total of 33 dogs with leishmaniosis were included in the study. Each dog was categorized at the time of diagnosis into I out of IV clinical stages on the basis of clinical signs, clinicopathological abnormalities, and serological status, according to published LeishVet guidelines [2].

Inclusion criteria were dogs clinically affected by *Leishmania*, with a serum cPLI concentration and abdominal ultrasound performed at the time of diagnosis and prior to treatment initiation (*T*_0_) and at the end of meglumine antimoniate treatment (*T*_30_), or dogs that presented with clinical signs compatible with pancreatitis at any time during the first month of treatment (*T*_*x*_).

Dogs were excluded if meglumine antimoniate treatment was given 30 days before study inclusion and if pancreatitis was diagnosed within 4 weeks before starting meglumine antimoniate treatment.

### Data and assays

Data recorded for all the dogs included age, body weight, sex, neuter status, previous clinical history, date of diagnosis, clinical signs, and drugs administered. Complete blood count (CBC), biochemistry, and urinalysis including urinary protein/creatinine ratio, were performed in all dogs as part of their routine follow-up evaluation at *T*_0_ and *T*_30_, or *T*_*x*_.

### Diagnosis of leishmaniosis

Leishmaniosis diagnosis in each dog was based on clinical signs, clinicopathological findings compatible with leishmaniosis, and confirmation by a positive quantitative serology, such as indirect fluorescent antibody technique (IFAT) or enzyme-linked immunosorbent assay (ELISA), and/or identification of *Leishmania* parasites by cytology, histopathology, or polymerase chain reaction (PCR).

### cPLI assay

Serum samples were submitted to a commercial laboratory (IDEXX Laboratories, Barcelona, Spain) for the assessment of cPLI concentration at *T*_0_, *T*_30_, or *T*_*x*_. The reference interval of the assay is 0–200 μg/l. A concentration ≥ 400 μg/l is considered highly suspicious for pancreatitis, while a concentration between 201–399 μg/l is in the questionable range and requires further clinical data, abdominal ultrasound findings, clinical signs compatible with pancreatitis, and exclusion of other pathologies to confirm or refute the diagnosis of pancreatitis [31].

### Abdominal ultrasound

Two ultrasound machines, Toshiba Aplio MX in hospital A in Valencia and Esaote MyLab Eight XP in hospital B in Barcelona were used in each center during this prospective study. Abdominal ultrasound examinations were performed by a board-certified veterinary radiologist or radiology resident-in-training under the direct supervision of a veterinary radiologist at *T*_0_ and *T*_30_ or *T*_*x*_. During each abdominal ultrasound, an ultrasonographic pancreatic assessment severity score (UPASS), ranging from 0–7, was calculated on the basis of evidence of pancreatic size, echogenicity, echotexture, echogenicity of surrounding mesentery, and peripancreatic free fluid. The higher the UPASS, the greater the ultrasonographic evidence of pancreatitis [32].

### Clinical diagnosis (CDx) of pancreatitis

A final CDx of pancreatitis was established in each dog at *T*_*x*_ or *T*_30_ by one of the internal medicine board certified authors after assessment of clinical information, including the clinical history and physical examination findings, clinicopathological data, and diagnostic imaging findings. In this study, a CDx of pancreatitis was based on exclusion of other pathologies, the presence of at least two compatible clinical signs of pancreatitis (vomiting, anorexia, abdominal pain, or lethargy) that had not been previously described at *T*_0_, and the following criteria: (1) serum cPLI concentration ≥ 400 μg/l irrespective of the UPASS score or (2) serum cPLI concentration between 200 and 400 μg/l associated with UPASS ≥ 4.

### Canine leishmaniosis treatment

Treatment of leishmaniosis consisted of a combination of subcutaneous meglumine antimonate (Glucantime®, Boehringer Ingelheim) at a dose of 100 mg/kg/24 h for 30 days, and oral allopurinol at 10 mg/kg/12 h for 1 year [33]. Additionally, the board-certified internal medicine authors could decide whether the addition of prednisone at a dose of 0.7 mg/kg/24 h for 7–15 days was indicated when there was a high suspicion of inflammation due to immune complex deposition in any organ associated with leishmaniosis [8].

### Statistical analysis

Data were analyzed using a commercial statistical software package (SPSS Statistics 26, IBM). Results for continuous data are expressed as their principal statistic parameters and represented graphically as box plots. Data were tested for normality using the Kolmogorov–Smirnov (*n* > 50), and the Shapiro–Wilk tests (*n* < 50). Chi-squared (*χ*^2^) test was used to examine the association between two categorical variables, such as the association between LeishVet clinical stage and CDx of pancreatitis; the use of prednisone and CDx of pancreatitis; sex and CDx of pancreatitis; age and CDx of pancreatitis; and an initial diagnosis or relapse of leishmaniosis and CDx of pancreatitis. Data that were not normally distributed were analyzed using the Mann–Whitney *U* test to compare continuous variables, such as the association between serum cPLI concentration and UPASS between the two timepoints. Due to the vast variability of breeds, the association of breed and CDx of pancreatitis could not be analyzed. For all analyses, statistical significance was set at a *p*-value of < 0.05, with a 95% confidence interval (CI).

## Results

### Animals

In total, 33 client-owned leishmaniosis-sick dogs, 18 from hospital A and 15 from hospital B, met the inclusion criteria and were enrolled into the study; 22 were male (12 entire and 10 neutered), and 11 were female (8 spayed and 3 entire). The median age of dogs enrolled was 7.2 years (range 11 months to 14 years). There were 19 breeds represented, including crossbreed (*n* = 8), Yorkshire Terrier (*n* = 3), American Staffordshire Terrier (*n* = 3), Spaniel Breton (*n* = 3), American Bully (*n* = 2), Border Collie (*n* = 2), Rat Terrier (*n* = 2), and 1 each of the following breeds: Rhodesian Ridgeback, Samoyed, Czech Wolfdog, Pitbull, Boxer, Labrador Retriever, Fox Terrier, Great Dane, Bullmastiff and Podenco. In addition, 20 dogs (20/33, 60.6%) were diagnosed with leishmaniosis for the first time and thirteen (13/33, 39.4%) were diagnosed with a relapse.

### Clinical signs, laboratory findings, and LeishVet clinical stage at diagnosis (*T*_0_)

The most common presenting complaints were lethargy (14/33, 42.4%); followed by dermatological lesions (10/33, 30.3%), including desquamative dermatitis (*n* = 2), ulcerative cutaneous granuloma (*n* = 1), nodular dermatitis (*n* = 1), generalized desquamation (*n* = 1), wound on the ear (*n* = 1), ulcerative dermatitis (*n* = 1), periocular alopecia (*n* = 1), ulcer on a paw (*n* = 1), exfoliative dermatitis and nasal ulceration (*n* = 1); generalized lymphadenopathy (8/33, 24.2%); lameness (8/33, 24.2%); anorexia/hyporexia (7/33, 21.2%); ocular signs (5/33, 15.1%), including uveitis (*n* = 4) and conjunctival granuloma (*n* = 1); weight loss (4/33, 12.1%); diarrhea (4/33, 12.1%); epistaxis (3/33, 9.1%); polyuria/polydipsia (2/33, 6%); vomiting (1/33, 3%); and fever (1/33, 3%).

The most common laboratory anomalies found were hypergammaglobulinemia (18/33, 54.5%), followed by proteinuria (14/33, 42.4%), anemia (10/33, 30.3%), increased liver enzymes (8/33, 24.2%), pancytopenia (3/33, 9.1%), thrombocytopenia (3/33, 9.1%), azotemia (3/33, 9.1%), neutropenia (1/33, 3%), hypoalbuminemia (1/33, 3%), hyperglycemia (1/33, 3%), and lymphocytosis (1/33, 3%). Additionally, eight dogs (8/33, 24.2%) did not have any laboratory abnormalities.

All dogs were included in one of the four clinical stages of LeishVet guidelines [2] on the basis of clinical signs, clinicopathological abnormalities, and serological status. No dog was included in stage I; 13 were included in stage II (13/33, 39.4%), 9 of these 13 in IIa and 4 in IIb; 11 in stage III (11/33, 33.3%); and 9 in stage IV (9/33, 27.3%). Further details of signalment, clinical signs, laboratory findings, first diagnosis or relapse, and Leishvet clinical stage of each dog are presented on Table 1.

**Table 1:** Signalment, clinical signs, laboratory findings, LeishVet stage, relapse, cPLI and UPASS, cDX of pancreatitis, use of prednisone, and clinical signs compatible with pancreatitis of all dogs included in the study

*T*30 (23 dogs) -> Dogs that didn't develop pancreatitis during antimoniate meglumine treatment												
Patient and hospital (A/B)	Signalment (age, sex, neutered status, breed)	Clinical signs	Laboratory findings	LeishVet stage	Relapse	Spec cPL *T*0 (μg/l)	UPASS *T*0	Spec cPL *T*30 (μg/l)	UPASS *T*30	cDx pancreatitis *T*30	Prednisone	Clinical signs (compatible with pancreatitis)
1 (A)	4, MN Podenco	Polyarthritis, diarrhea	Anemia, thrombocytopenia, hypergammaglobulinemia	3	YES	< 30	0	52	0	No	No	None
2 (A)	11, FN Spanish Breton	Weight loss, uveitis, diarrhea	Anemia, proteinuria	4	YES	< 30	0	< 30	0	No	No	None
3 (A)	7, FN, Rat Terrier	Ulcerative cutaneous granuloma, lymphadenomegaly, uveitis	None	3	YES	< 30	0	< 30	0	No	No	None
4 (A)	11, ME cross-breed	Epistaxis, generalized lymphadenopathy, lethargy	Hypergammaglobulinemia	2b	YES	260	0	310	0	No	No	None
5 (A)	10, ME Samoyed	Lethargy, hyporexia	Anemia, azotemia, proteinuria, increased ALKP, hypergammaglobulinemia	4	YES	< 30	0	507	0	Yes	No	Lethargy, vomiting
6 (A)	10, ME American Staffordshire Terrier	Lethargy, hyporexia, lameness	Anemia, lymphocytosis, hypergammaglobulinemia	3	NO	< 30	0	36	0	No	No	None
7 (A)	4, ME Czech wolfdog	Diarrhea, lymphadenomegaly, lethargy, anorexia	Anemia, elevation hepatic enzymes (ALKP, ALT, GGT), hypergammaglobulinemia	2a	NO	137	0	96	0	No	No	None
8 (A)	4, MN cross-breed	Desquamative dermatitis, uveitis, generalized lymphadenopathy	Hypergammaglobulinemia	2a	YES	< 30	0	< 30	0	No	No	None
9 (A)	6, MN Boxer	Interdigital nodule, desquamative dermatitis, generalized lymphadenopathy	Proteinuria, hypergammaglobulinemia	3	NO	44	0	68	0	No	No	None
10 (A)	10, FE Spaniel Breton	Polyarthritis, weight loss, lethargy	Hyperglycemia, elevation ALKP, hypergammaglobulinemia	3	YES	33	0	64	0	No	No	None
11 (A)	5, MN American Staffordshire Terrier	Nodular dermatitis, generalized lymphadenomegaly, lethargy, anorexia, weight loss	Pancytopenia	2a	NO	< 30	0	64	0	No	No	None
12 (B)	7, FN American Bully	Conjunctival granuloma	Pancytopenia, proteinuria, hypergammaglobulinemia	2b	NO	< 30	3	< 30	3	No	No	None
13 (B)	3, ME American Staffordshire Terrier	Generalized desquamation and lethargy	Azotemia, hypergammaglobulinemia	4	YES	94	2	271	3	Yes	No	Hyporexia, lethargy
14 (B)	3, FE, cross-breed	Wound on the ear	None	2a	YES	< 30	1	< 30	3	No	No	None
15 (B)	11, MN Pitbull	Anorexia, vomiting	Proteinuria, elevation ALKP, ALT	3	NO	< 30	4	316	5	Yes	Yes	Vomiting, lethargy
16 (B)	5, MN cross-breed	Exudative anterior uveitis	None	3	NO	99	4	83	3	No	No	None
17 (B)	9, FN, Great Dane	Polyarthritis	Anemia, thrombocytopenia, hypergammaglobulinemia	3	NO	< 30	3	< 30	0	No	Yes	None
18 (B)	3, MN American Bully	Lethargy	Proteinuria, increased ALT, hypoalbuminemia, hypergammaglobulinemia	4	NO	34	0	733	0	Yes	Yes	Abdominal pain, lethargy
19 (B)	1, ME Springer spaniel	Fever, epistaxis	Proteinuria, hypergammaglobulinemia	2b	NO	151	2	< 30	1	No	Yes	None
20 (B)	6, ME Bullmastiff	Weight loss	None	2a	NO	< 30	1	< 30	1	No	No	None
21 (B)	1, ME Border Collie	Polyarthritis	Pancytopenia, proteinuria, elevation ALKP	3	NO	230	3	57	3	No	Yes	None
22 (B)	6, FN, Rat Terrier	Ulcerative dermatitis	Anemia, neutropenia, hypergammaglobulinemia	2a	NO	< 30	1	< 30	0	No	Yes	None
23 (B)	1, MN Border Collie	Urinary incontinence, pu/pd	Proteinuria	4	NO	< 30	1	< 30	3	No	Yes	None

TX (10 dogs) → Dogs that developed pancreatitis during antimoniate meglumine treatment												
Patient	Signalment (age, sex, neutered status, breed)	Clinical signs	Laboratory findings	Leishvet stage	Relapse	Spec cPL *T*0 (μg/l)	UPASS *T*0	Spec cPL *Tx* (μg/l)	UPASS *Tx*	cDx pancreatitis *Tx*	Prednisone	Clinical signs (compatible with pancreatitis)
24 (A)	11, FE, crossbreed	Weakness, lethargy, anorexia	Anemia	2a	No	134	0	> 2000	2	Yes	No	Severe weakness, lethargy
25 (A)	8, ME, YST	Polyarthritis	Proteinuria, hypergammaglobulinemia	4	No	49	0	1534	2	Yes	No	Vomiting, diarrhea, pleural effusion
26 (A)	14, MN, YST	Periocular alopecia	Proteinuria, thrombocytopenia, hypergammaglobulinemia	3	Yes	198	2	1665	4	Yes	No	Anorexia, lethargy
27 (A)	3, FN Rhodesian ridgeback	Generalized lymphadenopathy, lethargy	Proteinuria, azotemia	4	Yes	115	0	> 2000	2	Yes	No	Vomiting, diarrhea, lethargy
28 (A)	13, FN cross-breed	Ulcer in a paw	None	2a	Yes	98	0	1546	0	Yes	No	Anorexia, lethargy
29 (A)	11, ME cross-breed	Epistaxis, generalized lymphadenopathy, polyarthritis, lethargy	Proteinuria, hypergammaglobulinemia	4	No	260	0	310	3	Yes	No	Anorexia, diarrhea
30 (A)	6, ME, YST	Exfoliative dermatitis, nasal ulceration	Elevation ALKP and ALT	2b	No	79	0	576	3	Yes	No	Anorexia, lethargy, vomiting
31 (B)	9, ME Labrador Retriever	Lethargy, hyporexia	Mild anemia	3	No	< 30	2	Death	5	Yes	Yes	Death
32 (B)	8, FN, Fox Terrier	Lethargy, pu/pd	Proteinuria, anemia, elevation ALKP, hypergammaglobulinemia	4	Yes	< 30	2	Death (euthanized due to hypovolemic shock)	7	Yes	No	Death
33 (B)	13, MN cross-breed	Diarrhea, lethargy, lameness	None	2a	Yes	89	2	636	0 (not visualized entirely)	Yes	Yes	Anorexia, vomiting, lethargy

*ALKP* alkaline phosphatase, *ALT* alanine aminotransferase, *CDX* clinical diagnosis, *cPLI* canine-specific quantitative pancreatic lipase immunoreactivity, *FE* entire female, *FN* neutered female, *GGT* gamma-glutamyl transferase, *ME* entire male, *MN* neutered male, *UPASS* ultrasonographic pancreatic assessment severity score, *YST* Yorkshire Terrier

### Clinical signs of pancreatitis during first month of treatment (*T*_*x*_)

In total, ten dogs (10/33, 30.3%) showed at least two clinical signs compatible with pancreatitis while treated with meglumine antimoniate (Glucantime®, Boehringer Ingelheim); seven (7/10, 70%) were from hospital A and three (3/10, 30%) were from hospital B. In addition, one dog showed clinical signs during the first week of treatment, five during the second week, and four during the third week. No dog experience compatible clinical signs during the fourth week of treatment.

Of these ten dogs, one died during the third week of treatment, and an abdominal ultrasound confirmed pancreatitis. Another dog who presented with hypovolemic shock and severe clinical signs compatible with acute pancreatitis was euthanized at the owners’ discretion. Although serum samples for cPLI could not be obtained from either dog premortem, both dogs were not excluded from the study because they had compatible clinical signs and abdominal ultrasound confirmed pancreatitis.

The most common clinical signs were lethargy/apathy (6/10, 60%), followed by anorexia (5/10, 50%), vomiting (4/10, 40%), diarrhea (3/10, 30%), sudden death (2/10, 20%), weakness (1/10, 10%), and pleural effusion (1/10, 10%).

Further details of the serum cPLI concentration and UPASS of each dog are presented on Table 1.

### Clinical signs of pancreatitis at the end of the treatment (*T*_30_)

Of the 23 dogs that did not have clinical signs compatible with pancreatitis during the first month of treatment, 4 dogs developed clinical signs compatible with pancreatitis upon reassessment a few days after the end of treatment (*T*_30_); 1 dog was from hospital A (1/4, 25%) and 3 dogs were from from hospital B (3/4, 75%). All dogs had apathy/lethargy (4/4, 100%), two had vomiting (2/4, 50%), one had hyporexia (1/40, 25%), and one had abdominal pain (1/4, 40%).

Further details of each dog’s clinical signs are presented on Table 1.

### Serum cPLI concentrations

At presentation (*T*_0_), 30 dogs (30/33, 90.1%) had a cPLI concentration < 200 μg/l, incompatible with pancreatitis; 3 (3/33, 9.1%) had a concentration between 200–400 μg/l, in the questionable range; and no dog had a concentration ≥ 400 μg/l, suggestive of pancreatitis. The mean serum cPLI concentration at *T*_0_ was 78.3 μg/l.

During the first month of treatment (*T*_*x*_), ten dogs (10/33, 30.3%) showed clinical signs compatible with pancreatitis. Of these, seven (7/10, 70%) had serum cPLI concentrations compatible with pancreatitis (≥ 400 μg/l); one (1/10, 10%) was in the questionable range (between 200–400 μg/l); and in two dogs a sample could not be obtained, as previously mentioned. The mean serum cPLI concentration at *T*_*x*_ was 1283.4 μg/l.

At the end of treatment (*T*_30_), serum cPLI was measured in 23 dogs that completed the treatment course. Of these, 18 (18/23, 78.3%) had concentrations < 200 μg/l, incompatible with pancreatitis; 3 (3/23, 13%) had concentrations in the questionable range (between 200–400 μg/l); and 2 (2/23, 8.7%) had concentrations compatible with pancreatitis (≥ 400 μg/l). The mean serum cPLI concentration at *T*_30_ was 128.6 μg/l.

Serum cPLI concentrations between the two timepoints (*T*_0_ and *T*_*x*_/*T*_30_) were statistically different (*p* = 0.002, 95% CI). Serum cPLI concentrations between *T*_0_ and *T*_*x*_ were statistically different (*p* = 0.012, 95% CI). However, serum cPLI concentrations between *T*_0_ and *T*_30_ were not statistically different (*p* = 0.124, 95% CI).

### Abdominal ultrasound and UPASS

Details of the abdominal ultrasound of each dog are presented in Table 2.

**Table 2:** Abdominal ultrasound findings of all dogs included in the study

*T*30 (23 dogs) -> Dogs that didn't develop pancreatitis during antimoniate meglumine treatment													
Patient and hospital (A/B)	US abnormalities	Pancreatic size	Pancreatic echogenicity	Pancreatic echotexture	Echogenicity surrounding mesentery	Peripancreatic free fluid	UPASS *T*0	Pancreatic size	Pancreatic echogenicity	Pancreatic echotexture	Echogenicity surrounding mesentery	Peripancreatic free fluid	UPASS *T*30
1 (A)	Biliary sludge, colelith	0	0	0	0	0	0	0	0	0	0	0	0
2 (A)	Hyperechoic hepatomegaly, biliary sludge, hypoechoic, heterogeneous spleen mass of 1.7 cm	0	0	0	0	0	0	0	0	0	0	0	0
3 (A)	Suspected gastric ulceration in the pyloric antrum; liquid content in the colon	0	0	0	0	0	0	0	0	0	0	0	0
4 (A)	Hepatomegaly, nodules and liver mass, hypoechoic nodules in the spleen	0	0	0	0	0	0	0	0	0	0	0	0
5 (A)	Hepatomegaly, chronic renal changes, bilateral pyelectasis, with isoechoic material in left renal pelvis Bilateral ureteral distention with thickened walls	0	0	0	0	0	0	0	0	0	0	0	0
6 (A)	Hepatic nodule, slightly heterogeneous splenic parenchyma	0	0	0	0	0	0	0	0	0	0	0	0
7 (A)	Hepatomegaly, incidental cyst in the right kidney	0	0	0	0	0	0	0	0	0	0	0	0
8 (A)	Hepatomegaly, nodules in the spleen, cortical mineralization in the kidneys	0	0	0	0	0	0	0	0	0	0	0	0
9 (A)	None	0	0	0	0	0	0	0	0	0	0	0	0
10 (A)	Hepatomegaly, chronic renal changes	0	0	0	0	0	0	0	0	0	0	0	0
11 (A)	None	0	0	0	0	0	0	1	0	0	0	0	0
12 (B)	Jejunal lymphadenopathy	1	1	1	0	0	3	1	1	1	0	0	3
13 (B)	None	1	1	0	0	0	2	1	1	1	0	0	3
14 (B)	Hepatomegaly, splenomegaly	0	1		0	0	1	1	1	1	0	0	3
15 (B)	None	1	2	1	0	0	4	1	2	1	1	0	5
16 (B)	None	1	2	1	0	0	4	1	2	0	0	0	3
17 (B)	Splenic mass, bilaterally cryptorchid	1	2	0	0	0	3	0	0	0	0	0	0
18 (B)	None	0	0	0	0	0	0	0	0	0	0	0	0
19 (B)	None	1	1	0	0	0	2	1	0	0	0	0	1
20 (B)	Hepatomegaly, splenomegaly	1	0	0	0	0	1	1	0	0	0	0	1
21 (B)	None	1	2	0	0	0	3	1	2	0	0	0	3
22 (B)	None	1	0	0	0	0	1	0	0	0	0	0	0
23 (B)	None	1	0	0	0	0	1	1	2	0	0	0	3

TX (10 dogs) -> Dogs that developed pancreatitis during meglumine antimoniate treatment													
Patient and hospital (A/B)	US abnormalities	Pancreatic size	Pancreatic echogenicity	Pancreatic echotexture	Echogenicity surrounding mesentery	Peripancreatic free fluid	UPASS *T*0	Pancreatic size	Pancreatic echogenicity	Pancreatic echotexture	Echogenicity surrounding mesentery	Peripancreatic free fluid	UPASS *Tx*
24 (A)	Hepatomegaly, abdominal lymphadenopathy, biliary sludge, splenic nodules with irregular margins	0	0	0	0	0	0	0	0	0	2	0	2
25 (A)	Hyperechoic hepatomegaly, biliary sludge, cholelithiasis, hyperechoic pancreas, distended colon with fluid content, enlarged adrenal glands	0	0	0	0	0	0	0	1	1	0	0	2
26 (A)	Hepatomegaly, gallbladder mucosal hyperplasia and cholelithiasis, 17 mm hypoechoic heterogeneous nodule in the spleen	0	1	1	0	0	2	1	1	1	1	0	4
27 (A)	Hepatomegaly, hyperechoic peritoneum adjacent to the caudal end of the right pancreatic lobe	0	0	0	0	0	0	0	0	0	1	1	2
28 (A)	Hepatic nodule, splenic myelolipomas, chronic renal changes	0	0	0	0	0	0	0	0	0	0	0	0
29 (A)	Hepatomegaly, heterogeneous splenic parenchyma	0	0	0	0	0	0	1	0	0	1	1	3
30 (A)	Hepatomegaly, heterogeneous splenomegaly	0	0	0	0	0	0	1	1	1	0	0	3
31 (B)	Splenomegaly with heterogeneous parenchima	1	1	0	0	0	2	1	2	0	2	0	5
32 (B)	Renal mineralizations, urinary sediment	1	1	0	0	0	2	1	2	1	2	1	7
33 (B)	Thickening of the duodenal wall with hypoechoic appearance, without differentiation of the wall layering, adjacent hyperechoic mesenteric fat. Multiple small intestinal bowels with increase echogenicity of the mucosal layer Lymphadenomegaly with hypoechoic appereance nad hyperechoic mesenteric fat Heterogeneous liver parenchyma with hypoechoic areas. Gallbladder sludge	1	1	0	0	0	2	0	0	0	0	0	0

*US* abdominal ultrasound, *UPASS* ultrasonographic pancreatic assessment severity score

At diagnosis (*T*_0_), 18 dogs had UPASS of 0 (18/33, 54.5%), 4 of 1 (4/33, 12.1%), 6 of 2 (6/33, 18.2%), 3 of 3 (3/33, 9.1%), and 2 of 4 (2/33, 6.1%). The mean UPASS at *T*_0_ was 1.

In addition, ten dogs developed clinical signs before the end of the treatment (*T*_*x*_) and ultrasound was performed in all of them. Of these, two had an UPASS of 0 (2/10, 20%), three of 2 (3/10, 30%), two of 3 (2/10, 20%), one of 4 (1/10, 10%), one of 5 (1/10, 10%), and one of 7 (1/10, 10%). The mean UPASS at *T*_*x*_ was 2.8.

At the end of treatment (*T*_30_), abdominal ultrasound was performed on 23 dogs, as 10 dogs had developed clinical signs compatible with pancreatitis prior to the end of treatment. A total of 14 had UPASS of 0 (14/23, 60.8%), 2 of 1 (2/23, 8.7%), 6 of 3 (6/23, 26.1%), and 1 of 5 (1/23, 4.3%). The mean UPASS at *T*_30_ was 1.08.

The UPASS between the two timepoints (*T*_0_ and *T*_*x*_/*T*_30_) was statistically different (*p* = 0.035, 95% CI). The UPASS between *T*_0_ and *T*_*x*_ was statistically different (*p* = 0.018, 95% CI). However, the UPASS between *T*_0_ and *T*_30_ was not statistically different (*p* = 0.886, 95% CI).

### CDx of pancreatitis

A total of 42.4% (14/33) dogs had a CDx of pancreatitis and 19 did not (19/33, 57.6%) on the basis of the presence or absence of clinical signs, serum cPLI concentration, and UPASS; ten dogs (10/33, 30.3%) at *T*_*x*_ and four dogs (4/33, 12.1%) at *T*_30_ developed pancreatitis. Neither sex nor age was statistically associated with the development of pancreatitis (*χ*^2^ = 2.789, d*f* = 6, *p* = 0.835, and *χ*^2^ = 5.589, d*f* = 2, *p* = 0.061, respectively). As previously mentioned, breed could not be included and analyzed in the model as possible association with CDx of pancreatitis due to the vast variability of breeds.

### LeishVet clinical stage of the dogs that developed pancreatitis

Of the ten dogs with CDx of pancreatitis at *T*_*x*_, three were classified as stage IIa (3/10, 30%), one as stage IIb (1/10, 10%), two as stage III (2/10, 20%), and four as stage IV (4/10, 40%). Of the four dogs with CDx of pancreatitis at *T*_30_, one was classified as stage III (1/4, 25%), and three as stage IV (3/4, 75%). Advanced LeishVet clinical stage was statistically associated with development of pancreatitis (*χ*^2^ = 6.362, d*f* = 2, *p* = 0.042).

### First diagnosis of leishmaniosis or relapse

A total of 20 dogs (20/33, 60.6%) were diagnosed with leishmaniosis for the first time and 13 dogs (13/33, 39.4%) were diagnosed with a relapse. Of those diagnosed for the first time, 8 dogs (8/20, 40%) developed pancreatitis and 12 (12/20, 60%) did not. For the dogs diagnosed with a relapse, six (6/13, 46.1%) developed pancreatitis and seven (7/13, 53.8%) did not. Neither the first diagnosis of leishmaniosis nor a relapse were statistically associated with the development of clinical pancreatitis (*χ*^2^ = 0.122, d*f* = 1, *p* = 0.727).

### Use of prednisone

A total of nine dogs (9/33, 27.3%) were prescribed prednisone by the internal medicine board-certified authors; two out of nine dogs were classified as LeishVet clinical stage IIa (2/9, 22.2%), one as stage IIb (1/9, 11.1%), four as stage III (4/9, 44.4%), and two as stage IV (2/9, 22.2%), and four out of nine dogs (4/9, 44.4%) had CDx of pancreatitis and were classified as LeishVet clinical stage IIa (1/4, 25%), stage III (2/4, 50%), and stage IV (1/4, 25%). The remaining five dogs (5/9, 55.5%) did not develop pancreatitis and were categorized into LeishVet stages IIa (1/5, 20%), IIb (1/5, 20%), III (2/5, 40%), and IV (1/5, 20%). The use of prednisone was not statistically associated with the prevention of clinical pancreatitis (*χ*^2^ = 1.317, d*f* = 2, *p* = 0.518).

### Treatment of pancreatitis

In the cases where pancreatitis was diagnosed during treatment, meglumine antimoniate and allopurinol were withdrawn, and supportive care was started with analgesia, antiemetics, and fluid therapy. Each case was then reassessed by one of the internal medicine board certified authors and allopurinol treatment was restarted.

## Discussion

Consistent with the clinical impression of veterinary practitioners in endemic areas, previous case reports, and research manuscripts in veterinary medicine [7, 18–22], this study reported that pancreatitis occurred in dogs with leishmaniosis treated with meglumine antimoniate. However, the significant percentage (42.4%) described in this study suggests that this adverse effect, or collateral consequence, is more common than previously reported in veterinary studies [7, 23], although it is consistent with studies in human medicine [13, 14, 16, 17]. This study suggests that these discrepancies between the presence and incidence of acute pancreatitis in dogs treated for leishmaniosis are a consequence of how the pancreatitis was diagnosed and the clinical stage of the dog at diagnosis.

There are some previous publications suggesting that pancreatitis in dogs could be triggered by *L. infantum* infection on the basis of the histological presence of different types of mild to moderate pancreatic inflammatory infiltrates [34–36] or the use of a clinicopathological marker of pancreatitis [22]. Due to the low parasite burden in pancreatic biopsies found in these dogs [34–36] and the small number of dogs evaluated with only one marker of pancreatitis [22], a cause-and-effect relationship between *L. infantum* and pancreatitis has not yet been established. However, despite previously published evidence, the possibility of leishmaniosis-induced pancreatitis was excluded in the present study, as none of the dogs included met the described criteria for CDx of pancreatitis at the time of diagnosis (*T*_0_). At *T*_0_, pancreatitis was excluded in all dogs with a cPLI in the questionable range (patients 4, 21, and 29, see Table 1) and in the dogs with a high UPASS (patients 15 and 16, see Tables 1 and 2), because they did not meet the described criteria for CDx of pancreatitis.

Confirmation or exclusion of acute pancreatitis in dogs is complex, and ultimately it is a clinical diagnosis made by the veterinary practitioner [25, 26, 28, 37]. To remove the ambiguity of making a diagnosis of pancreatitis, the criteria required were rigorously chosen and based on a combination of suspicious clinical signs (excluding other potential diseases with similar clinical presentations), serum cPLI concentration, and ultrasound findings [28]. The serum cPLI assay has immunoreactivity for pancreatic acinar cells [27, 38]. It has been refined by using monoclonal antibodies in a sandwich enzyme-linked immunosorbent assay (ELISA) and recombinant antigen for calibration, and now it is a commercially available assay. It has the highest sensitivity (21–71%) and specificity (100%) for detecting histopathologically confirmed pancreatitis [27, 32, 39–41]. However, the measurement of pancreatic lipase concentration has several limitations, as it can be elevated in some infectious diseases, intervertebral disc disease, foreign bodies, gastric dilatation and volvulus, and in some extra pancreatic diseases [28]. In dogs with azotemia or proteinuria, decreased renal excretion did not result in a consistent and correlated increase in the serum Spec cPL in dogs [42] nor in cats [43] and therefore was not deemed a limitation for the measurement of cPLI in the present study. Due to nonspecific clinical signs and the limitations of the cPLI assay, the use of abdominal ultrasound was important in this study to aid in the diagnosis of pancreatitis. Ultrasonographic findings consistent with acute pancreatitis include enlargement of the pancreas, hypoechoic areas within the pancreas, increased echogenicity of the surrounding mesentery, altered pancreatic echotexture, and dilatation of the pancreatic or biliary duct [32]. In this study, aiming to increase confidence in the diagnosis of pancreatitis, dogs were assessed for the presence of pancreatitis on the basis of clinical signs (excluding other diseases on the basis of physical examination and blood and urine analysis), measurement of serum cPLI, and abdominal ultrasound findings (based on the UPASS [32]). Nevertheless, most dogs diagnosed with pancreatitis in the present study had mild clinical signs, despite meeting the criteria for CDx of pancreatitis. In clinical practice when faced with this type of cases, it is uncommon to perform further investigations such as cPLI or abdominal ultrasound, and consequently, diagnosis of pancreatitis may be missed. This different diagnostic approach may explain why veterinary practitioners feel there is a low incidence of acute pancreatitis in these cases, and why it has only been described in a limited number of case reports [18–21]. In addition, it likely explains the difference between the results of this study and that from previous studies [7, 23], which did not include ultrasound assessment of the pancreas. Therefore, this study also reinforces the widely suggested fact that abdominal ultrasound is essential to reduce the likelihood of underdiagnosing pancreatitis [25, 28, 32, 37].

In addition to the fact that the previous study [23] did not perform an abdominal ultrasound, as mentioned previously, they also did not include any dog in LeishVet clinical stage IV, and only included 19 dogs in stage II and 1 in stage III. Interestingly, in the present study, only 4 out of 14 dogs diagnosed with acute pancreatitis were classified as stage II and the remaining 10 were more advanced stages (III or IV). This may suggest that dogs with advanced LeishVet clinical stages have a greater number of circulating immunocomplexes that can consequently produce inflammation, when being treated with antimonials, secondary to their deposition in different organs [44], similar to glomerulonephritis associated to canine leishmaniosis [8, 45, 46]. This difference between the percentage of dogs with LeishVet clinical stages III and IV could be another explanation for the higher percentage of pancreatitis in the present and other recent studies [7, 22], compared with an older study [23]. This could have been confirmed by histopathology [28], but due to the risk of the procedure and its invasive nature, it was not carried out. Unfortunately, it could not be performed in either of the two dogs that died, as it was declined by the owners.

Prevention of pancreatitis is challenging for clinicians, as there is not always a clear etiology [24, 25]. On the basis of the hypothesis that pancreatitis in this situation could be a consequence of the inflammation secondary to circulating immune complex deposition [44], the use of antiinflammatory dose of prednisone was thought to act similar to that when used for glomerulonephritis associated with canine leishmaniosis [8, 33]. Unfortunately, this study did not show a statistical association between the use of prednisone and the prevention of the development of pancreatitis. The lack of a significant difference may be due to the limited sample size of the study, as prednisone was only used in nine dogs in hospital B if there was a high suspicion of a large amount of circulating immune complexes. However, there was a trend that dogs treated with prednisone were less likely to develop pancreatitis, as six of the nine dogs that received prednisone were classified as advanced LeishVet clinical stages (III and IV), and only three of these developed pancreatitis. Therefore, future studies with a larger number of dogs with high-grade leishmaniosis treated with meglumine antimoniate and prednisone are needed to confirm this hypothesis. It is also not possible to suggest a protocol to prevent pancreatitis associated with the use of antimonials in dogs with leishmaniosis, nor what may be the best clinical decision on how to continue with the treatment of leishmaniosis in those dogs once pancreatitis is suspected or has resolved.

The present study suggests that acute pancreatitis is induced by meglumine antimoniate used to treat leishmaniosis in dogs, and it seems reasonable to monitor the pancreas during the first month of treatment. It also suggests that acute pancreatitis may be a collateral effect associated with the use of this drug in dogs with advanced clinical stage of leishmaniosis, rather than a direct adverse effect of the drug. Therefore, if clinical signs suggestive of pancreatitis are observed, it could be useful to recommend assessment of serum cPLI measurement and abdominal ultrasound, or if there is a high suspicion of the presence of circulating immune complexes because the dog has an advanced LeishVet clinical stage (III or IV).

Finally, this study had several limitations, such as its multicentric nature, with a relatively small number and variable signalment (age and breed) of dogs included, and the lack of a control group, as it would not be ethical to leave untreated dogs affected by leishmaniosis. Another limitation of this study was the lack of histopathology, which is considered the gold standard diagnostic method for the diagnosis of pancreatitis. The lack of histopathology makes it impossible to know the exact triggering cause of pancreatitis, its association with meglumine antimoniate, and the role of prednisone in its prevention. Not understanding the exact pathophysiological mechanism of pancreatitis caused by meglumine antimoniate also limits the clinical recommendations that veterinarians can make when pancreatitis is detected, such as reducing the dosage of meglumine antimoniate, discontinuing the treatment, or using other antileishmanial drugs that have no reported effects on the canine pancreas.

## Conclusions

Meglumine antimoniate remains the first-line leishmanicidal treatment option for canine leishmaniosis, but it appears to induce pancreatitis, particularly in a significant percentage of dogs with advanced LeishVet clinical stages. Monitoring serum cPLI levels and performing an abdominal ultrasound should be considered during the first month of treatment if potential clinical signs of pancreatitis are observed or in dogs with advanced clinical stages.

## Data Availability

No datasets were generated or analyzed during the current study.

## Abbreviations

CBC: Complete blood count
CDX: Clinical diagnosis
cPLI: Canine-specific quantitative pancreatic lipase immunoreactivity
ELISA: Enzyme-linked immunosorbent assay
IFAT: Indirect fluorescent antibody technique
*L. infantum*: *Leishmania infantum*
PCR: Polymerase chain reaction
UPASS: Ultrasonographic pancreatic assessment severity score
